# Protocol of a cost-effectiveness analysis of a combined intervention for depression and parenting compared with enhanced standard of care for perinatally depressed, HIV-positive women and their infants in rural South Africa

**DOI:** 10.1136/bmjopen-2023-082977

**Published:** 2024-08-01

**Authors:** Carmen Sue Christian, Lungiswa Nkonki, Chris Desmond, Cecilia Hoegfeldt, Samukelisiwe Dube, Tamsen Rochat, Alan Stein

**Affiliations:** 1 Department of Economics, Faculty of Economics and Management Sciences, University of the Western Cape, Bellville, South Africa; 2 Health Systems and Public Health, Faculty of Medicine and Health Sciences, Stellenbosch University, Stellenbosch, South Africa; 3 School of Economics and Finance, University of the Witwatersrand, Johannesburg, South Africa; 4 Department of Psychiatry, University of Oxford, Oxford, UK; 5 Africa Health Research Institute, Durban, South Africa; 6 Department of Psychology, Faculty of Health and Education, Manchester Metropolitan University, Manchester, UK; 7 SAMRC/Wits Developmental Pathways for Health Research Unit, University of the Witwatersrand Johannesburg, Johannesburg, South Africa; 8 MRC/Wits Rural Public Health and Health Transitions Research Unit (Agincourt), School of Public Health, Faculty of Health Sciences, University of the Witwatersrand Johannesburg, Johannesburg, South Africa

**Keywords:** Protocols and guidelines, Community child health, Depression & mood disorders, PUBLIC HEALTH, HEALTH ECONOMICS, Randomised Controlled Trial

## Abstract

**Introduction:**

Poverty, HIV and perinatal depression represent a triple threat to public health in sub-Saharan Africa because of their combined negative effects on parenting and child development. In the resource-constrained context of low-income and middle-income countries, a lay-counsellor-delivered intervention that combines a psychological and parenting intervention could offer the potential to mitigate the consequences of perinatal depression while also optimising scarce resources for healthcare.

Measuring the cost-effectiveness of such a novel intervention will help decision-makers to better understand the relative costs and effects associated with replicating the intervention, thereby supporting evidence-based decision-making. This protocol sets out the methodological framework for analysing the cost-effectiveness of a cluster randomised controlled trial (RCT) that compares a combined intervention to enhanced standard of care when treating depressed, HIV-positive pregnant women and their infants in rural South Africa.

**Methods and analysis:**

This cost-effectiveness analysis (CEA) protocol complies with the Consolidated Health Economic Evaluation Reporting Standards 2022 checklist. A societal perspective will be chosen.

The proposed methods will determine the cost and efficiency of implementing the intervention as per the randomised control trial protocol, as well as the cost of replicating the intervention in a non-research setting. The costs will be calculated using an appropriately adjusted version of the Standardised Early Childhood Development Costing Tool.

Primary health outcomes will be used in combination with costs to determine the cost per improvement in maternal perinatal depression at 12 months postnatal and the cost per improvement in child cognitive development at 24 months of age. To facilitate priority setting, the incremental cost-effectiveness ratios for improvements in child cognitive development will be ranked against six other child cognitive-development interventions according to Verguet *et al*’s methodology (2022).

A combination of activity-based and ingredient-based costing approaches will be used to identify, measure and value activities and inputs for all alternatives. Outcomes data will be sourced from the RCT team.

**Ethics and dissemination:**

The University of Oxford is the sponsor of the CEA. Ethics approval has been obtained from the Human Sciences Research Council (HSRC, #REC 5/23/08/17), South Africa and the Oxford Tropical Research Ethics Committee (OxTREC #31–17), UK.

Consent for publication is not applicable since no participant data are used in this protocol.

We plan to disseminate the CEA results to key policymakers and researchers in the form of a policy brief, meetings and academic papers.

**Trial registration details:**

ISRCTN registry #11 284 870 (14/11/2017) and SANCTR DOH-27-102020-9097 (17/11/2017).

**STRENGTHS AND LIMITATIONS OF THIS STUDY**

- The costing tool will be derived from the Standardised Early Childhood Development Costing Tool. This is a standardised, granular costing tool that permits a deeper analysis of the intervention and ensures that costing results are comparable across different contexts and interventions.
- We will use a recently developed framework by Verguet *et al* (2022) for the economic evaluation of ECD interventions, which has the advantage of enabling direct cost-effectiveness comparisons of interventions across a range of contexts and settings. We could not find a comparable methodology and rankings for maternal perinatal depression, and therefore, this analysis will not be repeated for the depression outcome.
- Outcome measurements in randomised controlled trials (RCTs) are more detailed than in reality, which may lead to findings that overstate the intervention’s cost-effectiveness when implemented at the population level. Therefore, we also estimate the cost of replicating the intervention in a non-research setting.
- Although considerable effort will be made to determine the extent to which participant management and resource use in the RCT reflects practice in a non-research setting, it is inevitable that protocol biases are likely to influence the findings. As is the case when analysing the cost-effectiveness of all RCTs, such protocol biases limit the generalisability of the study findings.

## Introduction

 The combined burden of poverty, HIV and depression during the perinatal period are experienced by up to a third of women in sub-Saharan Africa, and has become a growing public health concern.^1^ In addition to negatively impacting the mother’s health, perinatal depression has documented negative effects on parenting and key areas of child development, especially in poor communities. Although several studies show the benefits of psychological interventions for perinatal depression in low-income and middle-income countries (LMICs),^2 3^ none have assessed a home-based integrated psychological *and* parenting intervention for HIV-positive women using task sharing to lay counsellors. In the resource-constrained context of LMICs, a lay-counsellor delivered intervention—if effective—would have potential to help mitigate the effects of this important public health issue.

Based on this rationale, a cluster randomised controlled trial RCT) was designed to evaluate the effect of a home-based intervention for perinatally depressed HIV-positive women that integrated psychological treatment for depression with a parenting programme. The protocol for the RCT is available in Rochat *et al*here.^1^

In addition to establishing effectiveness of the intervention, the cost-effectiveness of the intervention will be determined as outlined in this cost-effectiveness analysis (CEA) protocol. Measuring the cost-effectiveness will help decision-makers to better understand the relative costs and effects associated with replicating the intervention, thereby supporting evidence-based decision-making when allocating resources.

This protocol strengthens the practice of sharing health economic evaluation methods that support replicable and comparable CEAs of RCT interventions because we propose using a standardised costing tool and a recently developed priority setting framework. It also keeps researchers and funding bodies abreast of future health economic evaluations of RCTs aimed at improving maternal depression and child cognitive development.

We aim to address the following research question in this protocol: what is the cost-effectiveness of a combined psychological and parenting intervention compared to enhanced standard of care (ESoC) when treating perinatally depressed, HIV-positive women with their infants in rural South Africa? To answer this question, the protocol sets the following objectives:

1. To determine the cost of implementing the RCT as per trial protocol.
2. To estimate the cost of replicating the intervention in a non-research setting.
3. To determine the cost-effectiveness of the intervention, specifically the cost per improvement in coprimary outcomes (maternal perinatal depression and child cognitive development).

## Methods and analysis

A short health economic analysis plan was developed by the trial economist as part of the original RCT protocol.^1^ Subsequently, a more detailed health economic evaluation protocol was developed by an independent consulting health economist. Some of the information that guided this more refined and detailed protocol was extracted from the RCT protocol.^1^

As far as possible, this protocol complies with the consolidated health economic evaluation reporting standards 2022 checklist (title, introduction and methods).^4^ The methods and analysis section describes the boundaries for the analysis (study population, setting and location, selected perspective of the economic evaluation and analytical time horizon); our understanding of the intervention and comparator, measuring effects, the costing approach, analysis of costs and effects; and suggested sensitivity analyses and potential study limitations. For each of the methods proposed, a rationale or justification is provided for the choice.

### Boundaries of analysis

The boundaries for analysis are summarised in table 1 and describe the different approaches and justification of choices for a range of analytical considerations. The objectives of the CEA were

**Table 1:** Summary of analysis boundaries

Issues for consideration	Approach chosen	Justification of choice
Objectives of analysis	**Cost analysis:** to calculate total financial and economic costs and cost-efficiency of the intervention.**Cost-effectiveness analysis:** to calculate cost per improvement in coprimary outcomes (maternal perinatal depression at 12 months postnatal and child cognitive development at 24 months of age) and to compare costs to a vector of benefits, including indicative estimates of long-term gains, such as improved education outcomes and increased income in adulthood.	These are the most feasible objectives for analysis, given the time and resource constraints.
Audience	Researchers, healthcare workers and policy makers	Health economic evaluation commissioned by trial team.
Viewpoint	Societal perspective	Useful to multiple stakeholders to determine appropriate resource allocation.
Time		
a)Time of the intervention	April 2018 to December 2023	As per updated RCT protocol, with delays in measuring coprimary outcomes due to the COVID-19 pandemic.
b)Time over which benefits experienced	Approximately third trimester until 24 months post partum.	Benefits should be realised from the start of therapy (third trimester) until the final coprimary outcomes are measured at 24 months post partum.
Analytical horizon	The outcomes time horizon will run from when the first coprimary outcomes are measured (2019) until the final coprimary outcomes are measured (2024), that is, 5 years.The costing time horizon will run from inception (2016) until all therapy sessions are completed (2023), that is, 8 years.	The benefits time horizon will be updated to ensure that it matches the costs time horizon.
Which alternatives could be used for comparison?	Enhanced standard of care (ESoC)	ESoC provided to participants in the control arm of the trial.
Target population(s)	Pregnant women who are diagnosed HIV-positive; aged 16 years and above (all participants were above 18); meet the criteria for antenatal depression as defined by a score of ≥9 on the Edinburgh Postnatal Depression Scale; live or are planning to live within the study area at the time of delivery and for at least 9 months after delivery (intensive therapy period); are conversant in isiZulu or English. Exclusion criteria may be found in Rochat *et al*here. in Rochat et al..^1^	HIV-positive women have a higher risk of developing perinatal depression and they and their infants suffer from worse consequences thereof.
Coverage	To be implemented at 88% (15/17) of clinics in the district.	These clinics are research sites for a research centre.
Type of analysis	Cost-effectiveness analysis	Cost and consequences data are collected for the interventions and comparator.

RCTrandomised controlled trial

specified by the trial economist in consultation with the independent consulting health economist and the full trial research team.

This CEA is regarded as a full health economic evaluation because (i) the costs will be determined and examined relative to outcomes, and (ii) at least one alternative will be compared with the ESoC.

A societal perspective will be chosen, so the costing boundaries include provider and user costs. This perspective is justified in more detail below.

HIV-positive, perinatally depressed pregnant women and their infants are the targeted population for this intervention because HIV-positive women have a higher risk of developing perinatal depression and suffer from worse consequences thereof.^5^,^8^ The intervention aims to promote both the health and well-being of the mother and child.

### Study population, setting and location

The inclusion criteria for the RCT study population (HIV-positive, perinatally depressed pregnant women) required that they live (or planned to live) within the study area at the time of delivery and for at least 9 months after, and were conversant in isiZulu or English (more details on the inclusion and exclusion criteria can be found in online supplemental table 1).

The final sample size of the study population is 320. A larger sample size of 528 was originally estimated in the RCT protocol. However, recruitment slowed substantially due to COVID-19 lockdowns and restrictions. When we reached a sample of 320 participants—following guidance from the Trial Steering Committee—the statisticians reviewed the sample size in lieu of the very low attrition (much lower than anticipated in the original sample size calculation). These calculations showed that the final sample had adequate power (over 80%) to detect the difference in primary outcomes between arms. It was then decided to close recruitment.

The RCT study setting is in Northern KwaZulu-Natal, South Africa, where the community is mainly rural but includes an urban township and informal peri-urban settlements. The RCT is implemented at 49 geospatial clusters in the area. The study population characteristics are well documented as it falls within a demographic and health surveillance area, and it has been established that participants are economically vulnerable.^9^ This socioeconomic context is important to note because it supports the perspective chosen for the economic evaluation.

### Description of the intervention and comparator

The intervention analysed in the RCT is a novel, home-based combination of two evidence-based interventions (i) behavioural activation (BA) for depression and (ii) a parenting programme, that promotes early childhood development, especially cognitive development.^1^

The comparator, which participants in the control arm received, is an enhanced standard of care (ESoC) modelled on the enhanced care package used in a recent perinatal depression RCT in South Africa.^10^

Women in both arms of the trial have access to primary healthcare and hospital-based mental health services.^1^

A summary description of the intervention and the ESoC is provided in online supplemental table 1). Having a thorough understanding of the processes involved in each alternative is an important step in the health economic evaluation because it informs the costing approach, particularly in identifying the appropriate costs.

The preparation for the RCT (including funding contracting, ethics approvals, participant engagement and stakeholder consultations, staff recruitment and training) began in 2016, and implementation started in 2018. A total of 155 participants were enrolled and cluster-randomised to the intervention arm of the RCT while 165 participants were enrolled and cluster-randomised to the ESoC comparator. All intervention therapy sessions, ESoC calls and assessment of the coprimary outcomes should be completed by October 2023. However, data will not be available for analysis until unblinding in August 2024.

The main service provider of the intervention is the lay counsellor who delivers 10 counselling sessions and a booster session to participants (11 sessions in total). Completion is defined as a participant taking part in at least six (out of 10) sessions, excluding the booster session. The main service provider for the ESoC is the ESoC caller who makes four calls (two antenatal and two postnatal) to participants in the control arm to provide telephonic counselling support and advice, which also ensures that participants are helped to access care and referrals are made where necessary. Completion is defined as a participant taking part in at least three out of four calls.

Due to restrictions on movement because of the COVID-19 pandemic, both the therapy manual and the delivery approach for the intervention arm were adapted. This pivot allowed the RCT to continue under severe constraints. Adaptations were made to deliver components of the BA therapy by telephone, and some sessions were conducted telephonically from April 2020. In-person therapy resumed partially in November and December 2020, and then stopped again until March 2021. Therefore, depending on the severity of lockdown restrictions, participants in the intervention arm received a blend of in-person and telephonic sessions. The core content of the intervention remained the same, however, there were some differences in the delivery of therapy relating to the parent–child interactions. This may have led to some differences in effects. However, all participants had at least one face-to-face session which was important to set-up the therapeutic relationship and only a small number had the majority of sessions delivered telephonically. Telephonic therapy was carefully monitored and fidelity checked. In contrast to the intervention, no changes to method of delivery occurred for ESoC during COVID-19 restrictions because it was conducted via telephone only.

### Perspective

A societal perspective is chosen for this economic evaluation plan because it considers all costs (both provider and user). As a result, a societal perspective is more helpful when determining the appropriate resource allocation. Also, the success and sustainability of any home-based intervention depends on provider *and* user costs. If the targeted users, perinatally depressed, HIV-positive mothers, experience the intervention as too costly (eg, time away from paid work), then an implicit barrier to the intervention is created impacting their health-seeking behaviour.

### Time horizon and discount rates

The analytical horizon for costing will be 7 years, starting from the intervention inception in 2016 up until the completion of therapy sessions/ESoC calls in 2023 (see table 1). The analytical horizon for effects will be 4 years. Although the benefits of the intervention may accrue from the start of the intervention (third trimester) until the final coprimary assessment, the coprimary outcomes are only measured from 2019 (12 months after the first participant received therapy) until the end of the RCT in 2023 (when the final coprimary outcomes are measured for the final participant). The benefits time horizon will be adjusted accordingly so that the time horizons for costs and benefits match.^11^

Since the intervention will result in both short-term and long-term health effects, we need to apply a discount rate to the costs and consequences to account for society’s time preference for immediate versus future effects. Because effects were measured at least 2 years after the intervention was received, it is prudent that present values are adjusted accordingly. A discount rate of between 3% and 5% is often used when doing present value analysis,^12 13^ but this parameter will be varied (0%–10%) when doing sensitivity analysis for both the cost and health-effect findings.

### Measuring effects (outcomes)

One of the key objectives of this economic evaluation plan is to calculate the cost per natural unit of effect (the unit in which the clinical effect is measured) for the intervention as part of the CEA. This is done by calculating the cost per improvement in the coprimary outcomes selected by the RCT team as per their protocol:^1^ maternal perinatal depression at 12 months postnatal and child cognitive development at 24 months of age. For a range of reasons—including pauses due to the COVID-19 pandemic—data on child cognitive development are collected up to 42 months of age in some cases. Fortunately, the tool used to measure child cognitive development, described below, can be adjusted for age.

The coprimary outcomes are measured using the Edinburgh Postnatal Depression Scale (EPDS) and the Bayley Scales of Infant and Toddler Development III (BSID-III) cognitive subscale. The EPDS provides a total score while the (BSID-III) cognitive subscale provides a composite score. The mean differences of the coprimary outcomes will be calculated by the RCT research team.

In line with the cost-efficiency objective of this evaluation, it would be useful to measure the following intermediate and immediate outcomes: the total number of participants who completed the intervention and the total number of sessions conducted. Measuring these outcomes will allow cost-efficiency to be determined (eg, average cost per participant who completed the intervention and average cost per session). The data source for intermediate and immediate health-outcome measures will be the RCT records.

### Costing approach

A combination of activity-based and ingredient-based costing approaches will be used^14^ to identify, measure and value activities and inputs for all alternatives. Key informant interviews and a desktop review of the RCT protocol will provide information on the key activities of the intervention (and ESoC). Information on the ingredients used in the intervention (and ESoC) will be obtained in three broad ways: (1) expenditure reviews, (2) primary data collection with implementation staff (key informant interviews) and (3) participants (questionnaires).

The costing distinguishes between trial costs (the cost of implementing the intervention in an RCT context) and replication costs. This implies that (i) we will first calculate the cost to deliver the intervention (start-up and operational) *as is*, excluding research costs (eg, costs associated with collecting data, writing and submitting ethics applications, etc). This will be known as the base case. Thereafter, (ii) we will estimate costs for an operational-only scenario (ie, replicating the intervention in a non-research setting).

We will estimate the latter cost of an operational-only scenario to address some of the protocol biases inherent in RCT data that may impact costs and limit the generalisability of the findings. Within a highly controlled RCT setting, the nature of the resources (type, quantity and price) used during implementation are likely to be more specialised and therefore more costly than in a non-research setting. To address these biases, each intervention activity identified in the base case will be re-evaluated when estimating the operational-only scenario to consider whether the nature of the resources identified should remain as is, or whether more realistic resource types, quantities and prices should be used instead. This approach could result in, for example, adjusting the cadre type, number of staff and salary associated with a particular activity for the operational-only scenario so that the estimated costs more closely align to a non-RCT setting.

The following activities will be categorised as research, and therefore excluded from the costing: RCT administration, data collection and measurement of clinical outcomes as per the RCT protocol (please note that targeting is *not* excluded because it has been identified as an essential activity when replicating the intervention in a non-research setting).

Online supplemental tables 3 and 4 show the input requirements identified for each activity. Activities will be classified as either start-up or operational. Start-up activities are generally those engaged in before implementing the intervention (2016–2017), while operational activities occur repeatedly during implementation of the intervention (2018–2023). The once-off amendments to intervention manuals and comparator scripts occurred in 2020 (after implementation) are included as start-up costs because it was a once-off activity and are regarded as once-off.

We have identified eight main activities for the intervention and ESoC namely: start-up; targeting (costs associated with identifying users for the intervention, which includes participant recruitment); supervision (of lay counsellors or caller, includes fidelity checks); on-going training; therapy or calls; monitoring (monitoring of serious adverse events and adverse events); programme management (activities that are directly involved with managing the programme implementation (eg, management activities of the trial coordinator, trial project manager and principal investigators)) and central management. Central management activities are not directly linked to a specific programme. That is, it is a cross-cutting function for many other programmes besides this RCT (eg, finance management, human resources management or data management), and therefore this activity will be costed as a percentage of the total costs (between 10% and 15%). For the rest of the activities (and subactivities), inputs for each need to be identified.

Both total financial and economic costs will be calculated. Financial costs represent the actual expenditure on resource inputs used for the intervention. In contrast, economic costs—a broader concept—recognise the opportunity cost of using resources, and are therefore of greater interest and used in all final analyses.

After identifying all the inputs associated with activities, these will be categorised as either capital or recurrent cost items. Capital costs are incurred only once (or rarely) and are linked to items that have useful life years of one or more. Because start-up activities are once-off, the costs of their associated inputs are treated as capital. Recurrent costs are linked to inputs consumed within a year and paid for or purchased repeatedly. Inputs for operational activities could be categorised as either once-off (capital) or recurrent.

Given the relatively long duration of the intervention, it is inevitable that there will be staff attrition. This will be noted and accounted for in the costing.

Targeting is a key component of the RCT and will also be an important activity when replicating the intervention in a non-research setting. Given concerns about stigma, poor knowledge and lack of access associated with both HIV and mental illness, especially in rural communities, targeting is critical to better identify beneficiaries. Roadshows, for example, assist with educating potential beneficiaries about maternal depression and the available healthcare services, and therefore we add them as a key operational activity. On the supply side, targeting activities included regular standardisation at participating clinics. During standardisation, clinical staff received information on maternal depression and the RCT project and were reminded to refer all HIV-positive women to the recruitment team.

*Participant costs* are important to consider, given the socioeconomic vulnerability of the targeted population. Therefore, the *explicit cost* (eg, time away from paid work to attend sessions; transport costs and childcare costs) and *opportunity cost* (eg, time away from unpaid productive activities to attend sessions) of participating in the intervention will be accounted for too. Because these costs were attached to each session, they are classified as recurrent cost, but on the demand side since they affect participants. A questionnaire for participants was administered during some (administered during at least four therapy sessions) therapy sessions (and during all the ESoC calls). Topics of interest included in the questionnaire were:

- Expenditure to take part in sessions (eg, childcare costs).
- Time spent participating in intervention (time with provider and time on own intervention tasks);
- Activities missed while with provider and while implementing own intervention tasks.
- Salary information (if employed).

These participant-cost data will be supplemented by data on employment status from the sociodemographic module of the assessments. We will conduct a descriptive analysis of participant-cost data to estimate a range of participant costs.

Economic costs include the value of resources for which there were no financial transactions, for example, the opportunity costs of diverted provider resources. To capture this opportunity cost, a simple interview schedule for implementation staff (or key informants) will be designed to collect data on what resources they used during their implementation.

Often, there will be joint costs where inputs are used for both implementation and research. To deal with this during the expenditure review of financial costs to the provider, a system to allocate expenditure to research or implementation will be designed. This will be done by informally categorising expenditure as mainly research, shared, or mainly intervention and applying a fixed allocation percentage in each case, before estimating the costs.

The parameters of the cost data collected will include the quantity, cost per unit, time (in percentage) and expenditure year. In cases where data values are missing, appropriate assumptions will be made with justifications.

Straight-line depreciation—where the market value of the cost is divided by its remaining useful life—will be used to annuitise the financial cost of capital goods. To calculate the economic cost of capital goods, the market value will be divided by an annualising factor derived from the remaining useful life years and a discount rate. The discount rate used for the costing analysis will be set according to the latest South African interest rate for government bonds. This practice is feasible, given that the government bond rate is a good proxy of the opportunity cost of capital. Data on the useful life years of capital goods will be sourced from the latest South African Revenue Service’s note on depreciation allowances.

### Currency, price date and conversion

All costs will be expressed in South African Rand (ZAR)—the local currency—and US dollars (US$)—to facilitate global comparisons. The years of expenditure for all relevant costs varied between 2016 and 2023. Total costs will be adjusted by the inflation conversion rate to account for inflation during these 7 years, using 2018 as the base year. The convention for costing analyses is to use the year for which the most cost data were available (2018 in this case) as the base year.

### Analysis of costs and effects

First, the total financial and economic costs of the intervention and comparator will be calculated using an appropriately adjusted version of the standardised ECD costing tool (SECT).^15^ SECT is a standardised, granular costing tool that permits a deeper analysis of the intervention. More importantly, using SECT adds methodological consistency to the costing analysis, which ensures that costing results are comparable across countries and interventions. Next, the cost efficiency of the intervention will be calculated by dividing the total economic cost by intermediate and immediate outputs (total number of participants who completed therapy and total number of sessions), in turn, to determine the unit (or average) costs.

After costing the intervention as is (irrespective of delivery ‘mode’), we will create simple models to determine the cost of telephonic only or in-person only scenarios.

**Figure 1:**
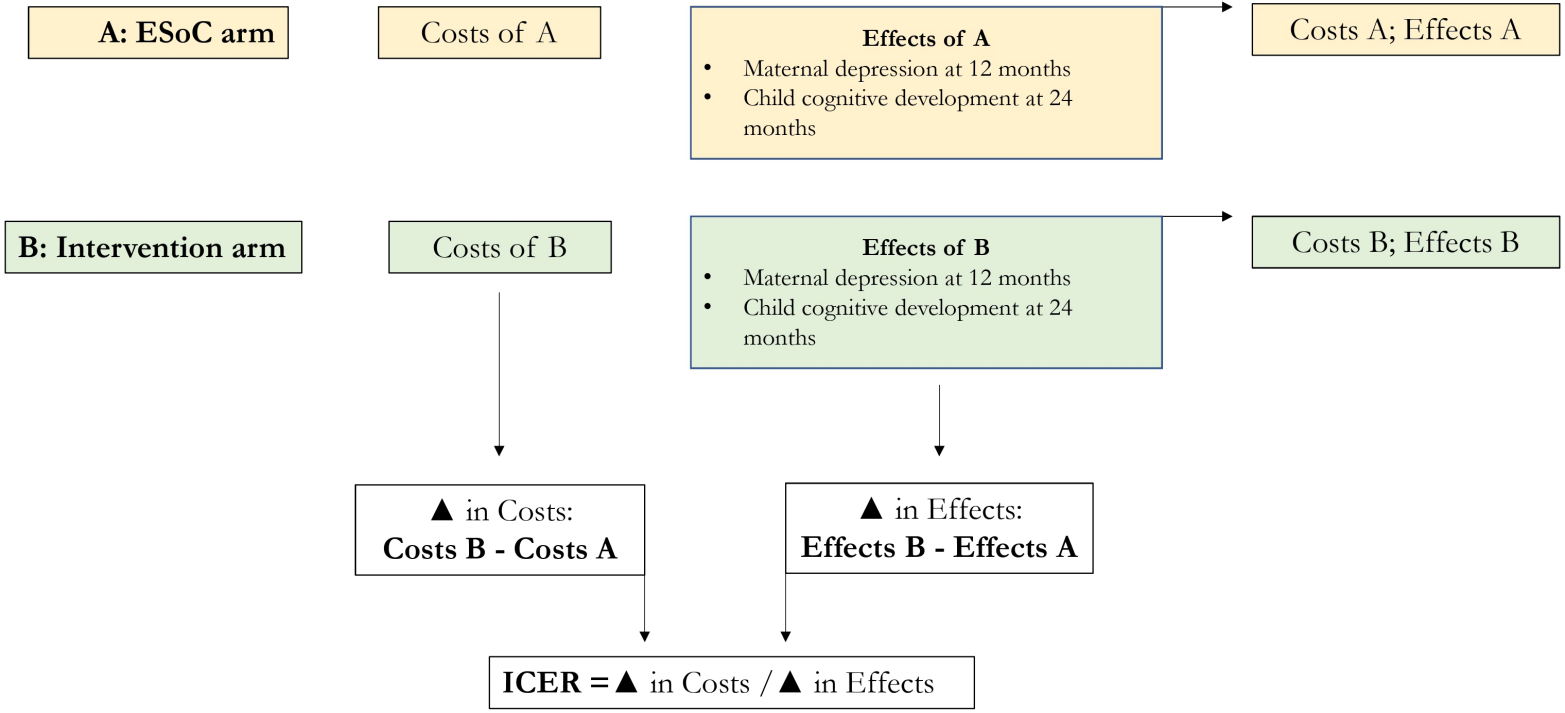
Basic CEA analysis flow chart. CEA, cost-effectiveness analysis; ESoC, enhanced standard of care.

Once all costs have been determined and analysed, we combine the effects to calculate the cost-effectiveness of the intervention. Figure 1 demonstrates the processes underlying the CEA. Changes in the costs and effects of the intervention, relative to the ESoC, will be used to calculate the incremental cost-effectiveness ratios. The flow chart underscores that incremental cost-effectiveness ratios (ICERs), and therefore cost-effectiveness, cannot be determined unless an intervention has proven to be effective relative to its comparator.

Primary health outcomes will be used in combination with costs to determine the following ICERs: cost per improvement in maternal depression at 12 months postnatal and cost per improvement in child cognitive development at 24 months of age.

After calculating the ICERs, we will replicate a recently developed framework that has been used for the economic evaluation of early childhood interventions, with the main purpose of facilitating priority setting.^16^ This framework has the advantage of enabling direct value-for-money (cost-effectiveness) comparison of interventions across a range of contexts and settings. While the original framework focuses on four dimensions of child development (motor skills, cognitive skills, language skills and socioemotional skills), we will rank the cognitive domain because the RCT only measures child cognitive development as a coprimary outcome (our CEA findings for maternal perinatal depression cannot be ranked using this tool).

To allow for comparison with other interventions that followed the same methodology, (replication) cost values will be standardised for LMICs by expressing the unit costs in 2010 US$ (using the World Bank’s consumer price index (2010=100) data). Available: https://data.worldbank.org/indicator/FP.CPI.TOTL?locations=US and using a wage reference point of US$3549 for 2010. Our ICERs for improvements in child cognitive development will then be ranked against six interventions that only measure child cognitive development (see table 2), as calculated by Verguet *et al* (2022).^16^

**Table 2:** Summary of estimated cost-effectiveness (ICER, 95% uncertainty ranges in parentheses) for each intervention that promotes ECD included in the Verguet *et al* (2022)^16^ study (cognitive domain only)

			LMIC standardised cost-effectiveness		Intervention cost-effectiveness (local cost)	
Study	Country	Effect estimate	Cost per child	ICER	Cost per child	ICER
Nair *et al*, 2009	India, 2008*	0.21 (0.06–0.35)	US$18	US$86 (US$51–US$300)	US$5	US$24 (US$14–US$83)
Aboud and Akhter, 2011	Bangladesh, 2008	0.40 (0.10–0.69)	US$99	US$248 (US$143–US$990)	US$69	US$172 (US$100–US$690)
Eickmann *et al*, 2003	Brazil, 1999	0.81 (0.46–1.16)	US$228	US$281 (US$197–US$496)	US$252	US$311 (US$217–US$548)
Nahar *et al*, 2009	Bangladesh, 2008*	0.84 (0.35–1.33)	US$582	US$693 (US$438–US$1663)	US$101	US$120 (US$76–US$289)
Vazir *et al*, 2013	India, 2012[Table-fn T2_FN1]	0.36 (0.14–0.57)	US$418	US$1161 (US$733–US$2986)	US$147	US$408 (US$258–US$1050)
Hamadani *et al*, 2006	Bangladesh, 2000–2002	0.33 (0.04–0.61)	US$1357	US$4112 (US$2225–US$33,925)	US$183	US$555 (US$300–US$4575)

* Study year not explicitly stated, so Verguet *et al* (2022)^16^ used the year prior to article publication.

LMIClow-income and middle-income countries

We could not find a comparable methodology and rankings for maternal perinatal depression, so the priority-setting exercise applied to child cognitive development outcomes will not be repeated for the depression outcome. Direct comparisons and rankings of depression outcomes for the few CEAs of interventions targeting perinatal depression^17^,^20^ are not advisable, as the contexts and designs of these studies are highly heterogeneous and uncertain, making it difficult to compare the cost-effectiveness across interventions or draw strong conclusions. We acknowledge this methodological limitation and, in future, hope to develop a priority-setting framework for interventions targeting perinatal depression outcomes.

### Sensitivity analysis

Sensitivity analyses will be conducted to address the impact of uncertainty on the economic evaluation findings, thus ensuring that the conclusions are robust in the study context and potentially generalisable to other settings. Using the Briggs *et al* (1994)^21^ taxonomy of uncertainty, the selected parameters and modelling approaches that will be subject to sensitivity analysis are summarised in table 3, with justification.

**Table 3:** Summary of parameters and modelling approaches subject to sensitivity analysis

Cause of uncertainty	Parameters and modelling approaches subject to sensitivity analysis	Reason
Data or sampling variations	Missing cost data	Some cost data collected retrospectively, so missing values will be assumed based on literature or comparable costs.
	Intervention delivery ‘mode’ costs	Unexpected pivot to telephonic sessions during COVID-19 pandemic meant that there is a wide variation in the ratio of in-person:telephonic sessions per participant.
Generalisability of results	Discount rates applied to costs and consequences	Key to explaining how costs and effects vary across different settings.
Methods used to measure and value costs and effects	Discounting (or not discounting) consequences Costing time horizon	Subject to disagreement among researchers, and greatly influence total effects. The costing time horizon will be adjusted accordingly so that the time horizons for costs and benefits match. This is in keeping with best practice.^4^

The range of values applied to sensitivity parameters or methods will be based on the literature and expert opinion.^22^ A simple approach to sensitivity analysis will be used, starting with univariate sensitivity analyses, before moving on to scenario sensitivity analyses. Once sensitivity analyses are complete, their findings will be interpreted in relation to the base-case results to determine robustness.^22^

### Limitations

While there are benefits to conducting a CEA alongside an RCT (eg, a lower marginal cost of collecting cost data alongside outcomes data), it also comes with disadvantages which may contribute to the limitations. Table 4 highlights some of the potential limitations, and their implications, relevant to this study.

**Table 4:** Summary of limitations and its implications

Limitation	Implication
Choice of comparator determined by the RCT	The RCT comparator, the ESoC, is not the *status quo* for treating perinatally depressed women in rural areas in South Africa. Normally, the most cost-efficient alternative (often the *status quo*) is selected as the comparator when conducting a CEA. This may limit the relevance of the findings to an academic audience rather than a broader one where policy decisions need to be based on more generalisable findings.
Outcomes measurements in RCTs are more detailed than in reality	This may lead to findings that overstate the intervention’s cost-effectiveness when implemented at a population level.
Protocol biases may impact both costs and outcomes	Although considerable effort will be made to determine the extent to which participant management and resource use in the RCT reflects practice in a non-research setting, it is inevitable that protocol biases are likely to influence the findings. As is the case when analysing the cost-effectiveness of all RCTs, such protocol biases limit the generalisability of the study findings.

CEAcost-effective analysisESoCenhanced standard of careRCTrandomised controlled trial

### Patient and public involvement (PPI)

There was no specific patient and public involvement (PPI) in the development of the health economics research question and its study design, but there was PPI in the development and design of the main trial.

## Ethics and dissemination

The University of Oxford is the sponsor of the trial and CEA. Ethics approval has been obtained from the Human Sciences Research Council (HSRC, #REC 5/23/08/17), South Africa, and the Oxford Tropical Research Ethics committee (OxTREC #31–17), UK.

Consent for publication is not applicable since no participant data are used in this protocol.

It was not appropriate or possible to involve patients and the public in the design of the CEA protocol. We do, however, plan to disseminate the CEA results (once available) to key policymakers and researchers in the form of a policy brief, meetings and academic papers.

### Trial registration details

ISRCTN registry #11 284 870 (14/11/2017) and SANCTR DOH-27-102020-9097 (17/11/2017).

## supplementary material

10.1136/bmjopen-2023-082977 online supplemental file 1
