# Supplementary material for: Protocol of a cost-effectiveness analysis of a combined intervention for depression and parenting compared with enhanced standard of care for perinatally depressed, HIV-positive women and their infants in rural South Africa
Source: BMJ Open. 2024 Aug 1;14(8):e082977. doi: 10.1136/bmjopen-2023-082977 (PMC11337707; doi:10.1136/bmjopen-2023-082977)
Supplement: online supplemental file 1 [file bmjopen-14-8-s001.pdf]

Supplemental Table 1: Summary of intervention and comparator descriptions

	**Combined psychological and parenting programme**	**Enhanced Standard of Care**
**Year of Inception**	2016	2016
**Sample size**	155 in intervention (48% of total trial sample)	165 enrolled in control ESoC arm (52% of total trial sample)
**Who? (Main contributors to the intervention should be described as this will help identify costs and sources of data needed for the evaluation)**	- Lay counsellors<br>- Risk-management nurse<br>- Trial psychologist<br>- Senior counsellors (lay counsellors with 12 months' experience of delivering interventions)<br>- Trial social worker<br>- National Department of Health (NDOH) psychologist<br>- Department of Social Development (DSD) social worker<br>- Trial coordinator<br>- Trial project manager<br>- Principal investigator: Psychiatrist<br>- Co-Principal investigator: Psychologist	- ESoC caller<br>- Risk-management nurse<br>- Trial psychologist<br>- ESoC supervisor (trial social worker/trial coordinator)<br>- Trial social worker<br>- National Department of Health (NDOH) psychologist<br>- Department of Social Development (DSD) social worker<br>- Trial coordinator<br>- Trial project manager<br>- Principal investigator: Psychiatrist<br>- Co-Principal investigator: Psychologist
**Does what? (Main activities associated with each intervention. This may include designing the clinical protocol, as well as any new training required in setting up an intervention or supervisory visits)**	***Lay counsellors*** deliver **10** counselling sessions and **one** booster session.<br>***Risk-management nurse*** assists with cases referred by trial psychologist.<br>***Trial psychologist*** provides training and supervision to counsellors. Manages patient cases and refers severe psychological cases as appropriate. Also oversees standardisation training at the clinics to help nurses identify potential participants.<br>***Senior lay counsellors*** provide therapy and weekly additional support to lay counsellors.<br>***Trial social worker*** monitors and assists with social welfare cases. Refers severe social welfare cases as appropriate.<br>***NDOH psychologist*** manages severe psychological cases referred by trial psychologist.<br>***DSD social worker*** manages severe social welfare cases referred by trial social worker.<br>***Trial coordinator*** is part of the sub-management group and oversees recruitment, assessment, therapy and ESoC activities. Also presents feedback to community advisory boards and the local hospital board.	***ESoC caller*** delivers information via four telephone calls.<br>***Risk-management nurse*** assists with cases referred by trial psychologist/ESoC supervisor.<br>***Trial psychologist*** provides training and manages patient cases and refers severe psychological cases as appropriate. Also oversees standardisation training at the clinics to help nurses identify potential participants.<br>***ESoC supervisor*** provides weekly supervision to ESoC caller.<br>***Trial social worker*** monitors and assists with social-welfare cases. Refers severe social-welfare cases as appropriate.<br>***NDOH psychologist*** manages severe psychological cases referred by trial psychologist.<br>***DSD social worker*** manages severe social-welfare cases referred by trial psychologist.<br>***Trial coordinator*** is part of the sub-management group and oversees recruitment, assessment, therapy and ESoC activities. Also presented feedback to community advisory boards and the local hospital board.

	Trial project manager is part of the management group and helped design the implementation strategy for the RCT intervention, lead certain trial components, and supported AE and SAE management and other operational tasks.<br>***Principal investigator (psychiatrist)*** is part of the management group that led the design of the RCT interventions and training manuals, supervises the trial psychologist and oversees AE and SAE management and referrals, and facilitates training.<br>***Co-Principal investigator (psychologist)*** is part of the management group that led the design of the RCT interventions and training manuals, provides training on perinatal mental-health management, supervises the trial psychologist and overseas adverse events and serious adverse events management and referrals, and facilitates training and debriefings.	***Trial project manager*** is part of the management group and helped design the implementation strategy for the RCT intervention, led certain trial components, and supported AE and SAE management and other operational tasks.<br>***Principal investigator (psychiatrist)*** is part of the management group that led the design of the RCT interventions and training manuals, supervises the trial psychologist and oversees AE and SAE management and referrals, and facilitates training.<br>***Co-Principal investigator (psychologist)*** is part of the management group that led the design of the RCT interventions and training manuals, provides training on perinatal mental-health management, supervises the trial psychologist and overseas adverse events and serious adverse events management and referrals, and facilitates training and debriefings.
**To whom?**	HIV-positive, perinatally depressed, pregnant women who are at 23–33 weeks of gestation at the time of enrolment. Post-partum, the infant is included in the intervention.	Same target group
**Where?**	In-person at participant's home (or telephonically)	Telephonic only
**How often?**	**11 sessions** held from two weeks post enrolment (antenatally) until nine months postnatal, along with an additional booster session at 16 months postnatal. Sometimes, sessions three and four of the intervention could only be delivered postpartum (cases of early deliveries or pregnancy complications which caused the mother to be hospitalised).<br>**Length of session:** 90 minutes	**Four calls** held from two weeks post enrolment (antenatally) until four months postnatal. Sometimes, the ESoC call two could only be delivered postpartum (cases of early deliveries or pregnancy complications which caused the mother to be hospitalised).<br>**Length of call**: 10-20 minutes

Supplemental Table 2: Description of outcome variables

Type of Outcome	Variable	Measure and metric	Data source
**Primary**<br>(***final effect on health status***)	Maternal depression at 12 months<br><br>Child cognitive development at 24 months	(EPDS), total score; mean difference<br><br>(BSID-III) cognitive subscale, composite score; mean difference	Secondary data from RCT team
**Intermediate**<br>***(changes before there is a health impact*****)**	Participants who completed intervention	Total number for each intermediate outcome	RCT records
**Output**<br>(***aka process or immediate***)	Sessions conducted	Total number for each output	RCT records

Supplemental Table 3: Description of intervention activities with respect to input requirements identified

**Activity**	**Required inputs**
**1. Start-up**	
1.1 Intervention design	Meeting venues, investigators' time
1.2 Development of intervention training manual and other materials	Meeting venues, investigators' time
1.4 Training manual adjustments to accommodate COVID-19	Meeting venues, investigators' time
1.4 Development of implementation strategy	Meeting venues, investigator's time (trial project manager)
1.5 Intervention pilot	Therapy materials (electronic tablets), work resources (vehicles, mobile phone), travel time to and from the households (for counsellors), utilities (data, petrol), participants' materials (pens, plastic folders), participant incentives (airtime), personnel time (counsellors), participant's time (time away from unpaid productive activities for attending sessions)
1.5 Recruitment of lay counsellors, senior counsellors (supervisors), and clinical psychologist	Venue, personnel time, investigators' time
1.6 Initial training of lay counsellors and senior counsellors (supervisors)	Venue, training materials, refreshments, personnel time (lay counsellors, senior counsellors, clinical psychologist), investigators' time
1.7 Initial community-engagement roadshow	Venue, refreshments, investigator's time (trial coordinator)
**Operational**[a]	
**2. Targeting**	

[a] Excludes central management costs which are calculated as a percentage of the total costs.

2.1 Community-engagement roadshows	Work resources (venue, vehicles), utilities (petrol), participation prizes (gift baskets), educational material, personnel time (ARHI community-engagement team)
2.2 Community advisory board and district hospital feedback	Work resources (venue, vehicles), utilities (petrol), personnel time (coordinator, ARHI nurse, occasionally the PI)
2.3 Intervention awareness (standardisation training) at participating clinics	Work resources (venue, vehicles), utilities (petrol), participation prizes (chocolates), personnel time (coordinator, senior counsellor, psychologist)
2.4 Participant screening and recruitment	Venue, screening materials and equipment, refreshments, personnel time, time of investigators, participant's time (direct costs, indirect costs, time away from unpaid productive activities for attending sessions)
**3. Supervision (of lay counsellors)**	Work resources (office, furniture, laptops, mobile phones), utilities (data, airtime, office supplies), personnel time (senior counsellor, trial psychologist)
**4. Ongoing training**	Work resources (office, furniture, laptops), training materials, refreshments, personnel time (clinical psychologist), investigators' time
**5. Therapy**	Therapy materials (electronic tablets), work resources (vehicles, mobile phone), utilities (data, petrol), travel time to and from the households (for counsellors), participants' materials (pens, plastic folders), participant incentives (airtime), personnel time (counsellors), participant's time (time away from unpaid productive activities for attending sessions)
**6. Monitoring**	Work resources (laptops, PCs), personnel time, investigators' time
**7. Programme management**	Work resources (laptops, PCs), personnel time, investigators' time , trial manager and trial coordinator.

Supplemental Table 4: Description of ESoC activities with respect to input requirements identified

Activity	Required inputs
**1. Start-up**	
1.1 ESoC design	Meeting venues, investigators' time
1.2 Development of ESoC training manual and other materials	Meeting venues, investigators' time
1.3 Training manual adjustments to accommodate COVID-19	Meeting venues, investigators' time
1.4 Development of implementation strategy	Meeting venues, investigator's time (trial project manager)
1.5 ESoC pilot	Call script (paper), work resources (mobile phone), utilities (data), participant incentives (airtime), personnel time (ESoC caller), participant's time (time away from unpaid productive activities for attending sessions)
1.6 Recruitment of lay caller, social worker (supervisor) and clinical psychologist	Venue, personnel time, investigators' time
1.7 Initial training of lay caller and social worker (supervisor)	Venue, training materials, refreshments, personnel time (lay counsellors, senior counsellors, clinical psychologist), investigators' time
1.8 Initial roadshow	Venue, refreshments, personnel time (therapists)
**Operational**[b]	
**2. Targeting**	
2.1 Community-engagement roadshows	Work resources (venue, vehicles), utilities (petrol), participation prizes (gift baskets), educational material, personnel time (therapists)
2.2 Community advisory board and district hospital feedback	Work resources (venue, vehicles), utilities (petrol), personnel time (coordinator, ARHI nurse, occasionally the PI)

[b] Excludes central-management costs which are calculated as a percentage of the total costs.

2.3 ESoC awareness (standardisation) at participating clinics	Work resources (venue, vehicles), utilities (petrol), participation prizes (chocolates), personnel time (coordinator, senior lay counsellor, psychologist)
2.4 Participant screening and recruitment	Venue, screening materials and equipment, refreshments, personnel time, investigators' time, participant's time (time away from unpaid productive activities for attending sessions)
**3. Supervision (of ESoC caller)**	Work resources (office, furniture, laptops, mobile phones), utilities (data, airtime, office supplies), personnel time (senior lay counsellor)
**4. Ongoing training**	Work resources (office, furniture, laptops), training materials, refreshments, personnel time (clinical psychologist), investigators' time
**5. Calls**	Call script (paper), work resources (mobile phone), utilities (data), participant incentives (airtime), personnel time (ESoC caller), participant's time (time away from unpaid productive activities for attending sessions)
**6. Monitoring**	Work resources (laptops, PCs), personnel time, investigators' time
**7. Programme management**	Work resources (laptops, PCs), personnel time, investigators' time (trial manager and trial coordinator)
