# Supplementary material for: Protocol of a cost-effectiveness analysis of a combined intervention for depression and parenting compared with enhanced standard of care for perinatally depressed, HIV-positive women and their infants in rural South Africa
Source: BMJ Open. 2024 Aug 1;14(8):e082977. doi: 10.1136/bmjopen-2023-082977 (PMC11337707; doi:10.1136/bmjopen-2023-082977)
Supplement: Supplementary file 2 [file bmjopen-2023-082977.reviewer_comments.pdf]

PEER REVIEW HISTORY

BMJ Open publishes all reviews undertaken for accepted manuscripts. Reviewers are asked to complete a checklist review form (**http://bmjopen.bmj.com/site/about/resources/checklist.pdf**) and are provided with free text boxes to elaborate on their assessment. These free text comments are reproduced below.

ARTICLE DETAILS

**TITLE (PROVISIONAL)**	Protocol of a cost-effectiveness analysis of a combined intervention for depression and parenting compared with enhanced standard of care for perinatally depressed, HIV-positive women, and their infants, in rural South Africa
**AUTHORS**	Christian, Carmen; Nkonki, Lungiswa; Desmond, Chris; Hoegfeldt, Cecilia; Dube, Samukelisiwe; Rochat, Tamsen; Stein, Alan

VERSION 1 – REVIEW

**REVIEWER**	Dadi, A.F.<br>Menzies School of Health Research, Centre for Child Development and Education
**REVIEW RETURNED**	21-Dec-2023

**GENERAL COMMENTS**	I would like to appreciate authors for dealing with such an important but under considered area of maternal health. The protocol addressed the what and the how questions of the cost effectiveness analysis of the combined interventions Vs the available stand of care in delivering health care for perinatally depressed HIV-positive women. The protocol is clear and complete, except the following minor edits.<br>1. It seems that all the data required to make this analysis is already available, and the protocol needs to address why publishing the protocol at this stage is required. The cost effectiveness analysis could have been part of the trial protocol.<br>2. The consistency of objectives, the research questions highlighted in lines from 127 to 129 includes infant population but the others only mentioned the women. This needs to be clarified.<br>3. "A primary outcome refers to the final effect of the intervention on the mental-health status of the participants (mother and child, in this case)"- consistency as above. The authors need to revise the title that the population of study also includes infants, but you also mentioned 24 to 42 months down the page as outcome--> you need to be consistent.<br>4. Line 258 "maternal perinatal depression at 12 months postnatal"- does this assess maternal depression both in pregnancy and postnatal period or only postnatal period? if this is only postnatal period it is better to say postnatal depression not perinatal depression.<br>5. Lines 412 to 414 "We could not find a comparable methodology and rankings for maternal perinatal depression, so the priority-setting exercise applied to child cognitive development outcomes will not be repeated for the depression outcome. If this is the case, why it is not possible to remove this objective from the protocol and analysis?<br>6. Generally, the protocol could be shortened by just focusing on cost-effectiveness analysis.

REVIEWER	Bhat, Amritha<br>University of Washington, Department of Psychiatry and Behavioral Sciences
REVIEW RETURNED	21-Feb-2024

GENERAL COMMENTS	Well written description of the study protocol. One minor modification: The authors state that " effectiveness will not be determined by the delivery mode". This bears more discussion as an intervention that calls for observation of parent child interaction will very likely have different effects based on whether it is delivered in the home or remotely.

VERSION 1 – AUTHOR RESPONSE

Response to Reviewer 1 (Dr Dadi) comments:

1. It seems that all the data required to make this analysis is already available, and the protocol needs to address why publishing the protocol at this stage is required. The cost effectiveness analysis could have been part of the trial protocol.

    - Thank you for raising this point. All the data were not available at the time of writing this protocol. The unblinded data needed for the effectiveness analysis will only be available from August 2024. All cost data were only released in 2024. Due to these factors, the cost-effectiveness analysis has yet to begin.

2. The consistency of objectives, the research questions highlighted in lines from 127 to 129 includes infant population but the others only mentioned the women. This needs to be clarified.

   - Thank you for highlighting this inconsistency. The title, abstract and manuscript and relevant tables have been updated to include the infant population.

3. "A primary outcome refers to the final effect of the intervention on the mental-health status of the participants (mother and child, in this case)"- consistency as above. The authors need to revise the title that the population of study also includes infants, but you also mentioned 24 to 42 months down the page as outcome--> you need to be consistent.

   - Thank you for picking this up. The title has been updated to include the infant. The infant has also been included in the study population.

   - The inconsistency regarding the 24 and 42 months has been rectified in the manuscript (page 7, line 265; page 11, line 402) and the figure.

4. Line 258 "maternal perinatal depression at 12 months postnatal"- does this assess maternal depression both in pregnancy and postnatal period or only postnatal period? if this is only postnatal period it is better to say postnatal depression not perinatal depression.

   - The depression at 12 months postnatal will be compared to the maternal perinatal depression assessed at recruitment. However, we do understand that the phrasing reads ambiguously. For this reason, we have updated the sentence to read: '…maternal depression at 12 months postnatal' (see page 11, line 401).

5. Lines 412 to 414 "We could not find a comparable methodology and rankings for maternal perinatal depression, so the priority-setting exercise applied to child cognitive development outcomes will not be repeated for the depression outcome. If this is the case, why it is not possible to remove this objective from the protocol and analysis?

   - Thank you for this suggestion. After careful consideration, we still think that estimating the ICER for the depression outcome would contribute to the scarce literature (even if we cannot rank in a priority-setting exercise similar to child cognitive outcomes).

6. Generally, the protocol could be shortened by just focusing on cost-effectiveness analysis.

   - Thank you for this advice. It has been taken into consideration and the following lines have been deleted:

   - Lines 106-115; Lines 199-200; Lines 204-209

Response to Reviewer 2 (Dr Bhat) comment:

1. Well written description of the study protocol. One minor modification: The authors state that " effectiveness will not be determined by the delivery mode". This bears more discussion as an

intervention that calls for observation of parent child interaction will very likely have different effects based on whether it is delivered in the home or remotely.

- You raise a valid point, thank you. We agree with this which is why home visits were chosen as the modality for therapy. This is because face-to-face therapy is more naturalistic and the observed interactions are more likely to be representative of the actual mother-infant interactions on a daily basis. This is important because it allows the therapist to provide real-time feedback. As mentioned in the protocol, we had to switch to telephonic therapy during the main COVID-19 lockdowns. Telephonic therapy was carefully monitored, and fidelity checked. Around 20% of all sessions were conducted telephonically during this period. Most of these sessions were postnatal therapy sessions. Important to note, however, the trial does not include any direct observational assessments of mother-infant interactions.
- The following revisions have been made to the manuscript to address this:
- Page 6, lines 224-229: "The core content of the intervention remained the same, however, there were some differences in the delivery of therapy relating to the parent-child interactions. This may have led to some differences in effects. However, all participants had at least one face-to-face session which was important to set up the therapeutic relationship and only a small number had the majority of sessions delivered telephonically. Telephonic therapy was carefully monitored, and fidelity checked."
